# Intrahepatic cholangiocarcinoma mimicking a liver abscess

**DOI:** 10.1002/ccr3.3201

**Published:** 2020-08-03

**Authors:** Haythem Yacoub, Hajer Hassine, Seif Boukriba, Slim Haouet, Asma Ayari, Hela Kchir, Nadia Maamouri

**Affiliations:** ^1^ Gastroenterology B Department La Rabta Hospital Tunis Tunis Tunisia; ^2^ Faculty of Medicine of Tunis El Manar University Tunis Tunisia; ^3^ Radiology Department La Rabta Hospital Tunis Tunis Tunisia; ^4^ Pathology Department La Rabta Hospital Tunis Tunis Tunisia

**Keywords:** intrahepatic cholangiocarcinoma, liver abscess, liver cancer

## Abstract

Intrahepatic cholangiocarcinoma masquerading as liver abscess, and presenting with fever, is a very rare situation and should be considered in nonresolving liver abscess. Only few cases were reported in the literature. This entity is characterized by late diagnosis and poor prognosis.

## Graphical abstract

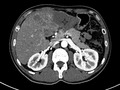

## 1 INTRODUCTION

Intrahepatic cholangiocarcinoma is characterized by late diagnosis and poor prognosis.^1^ Despite progress in radiological imaging techniques, obtaining a precise diagnosis in certain cases of liver tumors especially for ICC is not always allowed. Various imaging features may be observed in ICC.^2^ Typical computed tomography scan (CT scan) findings are thin peripheral arterial enhancement during the early phase, persistent enhancement during the delayed phase, and progressive centripetal filling.^3^

In rare cases, cholangiocarcinoma may mimic pyogenic liver abscess with symptoms like abdominal mass, fever, and leukocytosis upon presentation. Here, we report a case of an unusual first presentation of ICC with histological confirmation after an immunohistochemistry study, which was difficult to diagnose because of a radiological aspect mimicking a liver abscess on the enhanced contrast abdominal CT scan.

## 2 CASE REPORT

A 43‐year‐old man with a history of long‐standing alcohol consumption was admitted in our gastroenterology department due to abdominal pain located in the epigastric region with a history of intermittent fever and weight loss but not associated with any chills or night sweats. He denied any drug consumption, vomiting, jaundice, or pruritus. On examination, the temperature was 38°C, the abdomen was tender on palpation of upper quadrants, a 2‐cm tender hepatomegaly, blood pressure was 120/80 mm Hg, and pulse was 85 bpm.

Liver function tests showed serum aspartate aminotransferase (AST) levels about 2 times the upper limit of normal. Gamma‐glutamyl transferase (GGT) and alkaline phosphatase (ALP) levels were, respectively, 2 times and 3 times the upper limit of normal. Alanine aminotransferase (ALT) and total bilirubin levels were normal. A moderate increase of leukocytes was noted (13 600 E/mm^3^). C‐reactive protein (CRP) level was high (131 mg/L). Other parameters including hemoglobin, platelet count, renal function tests, and serum electrolytes were normal. Serum test for human immunodeficiency virus (HIV) and hepatitis C virus was negative. Hepatitis B markers were positive for HBs antigen; HBc and HBs antibodies were also positive. The HBs antibodies level was about 11 IU/L. Hepatitis B viral load was positive (105 IU/mL).

Upper gastrointestinal endoscopy was performed, and it was normal. Abdominal ultrasound showed a normal gallbladder without signs of cholecystitis and multiple left lobe liver masses.

**FIGURE 1:**
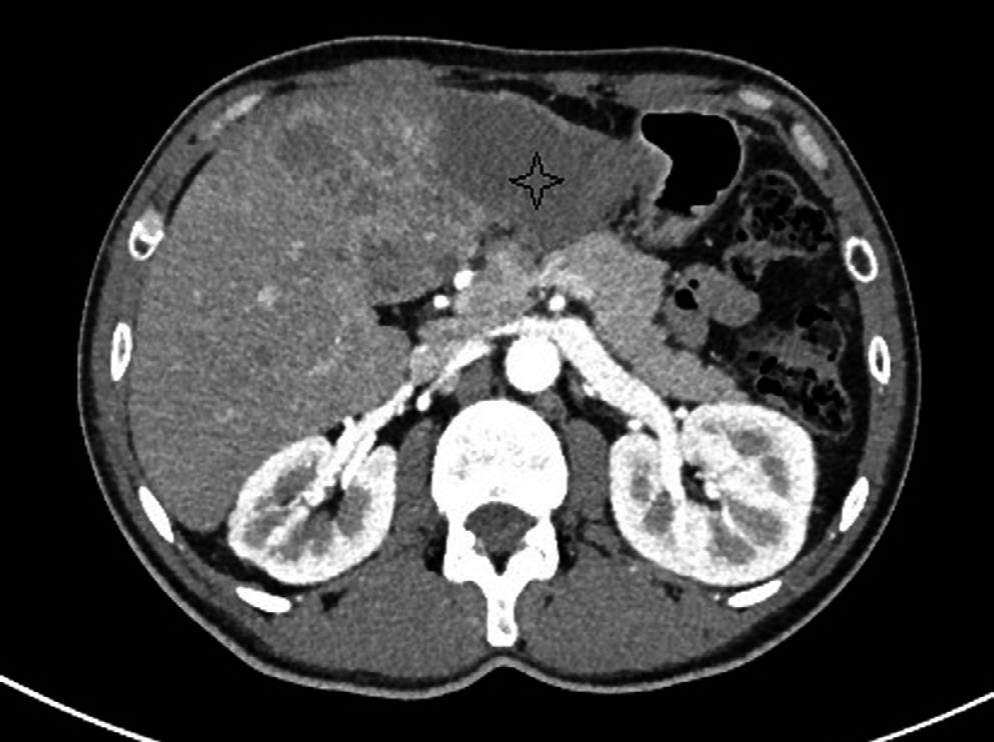
Arterial phase: Rim‐enhancing and centrally hypodense lesion (asterisk)

**FIGURE 2:**
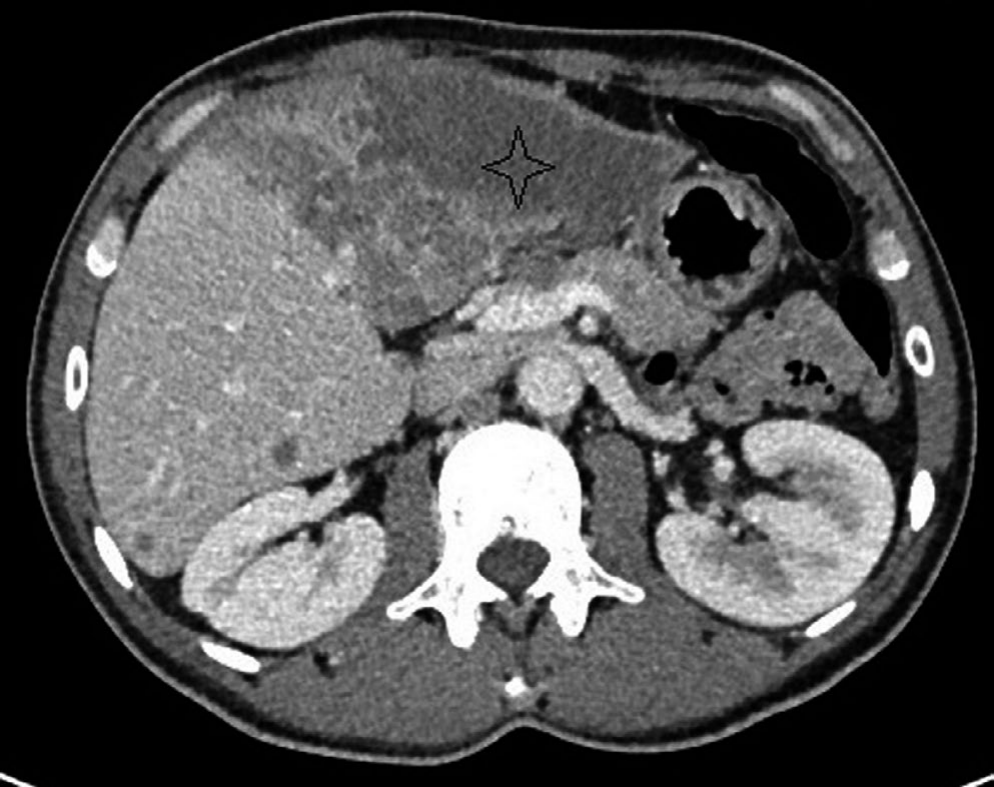
Portal phase: hypovascular nodular lesions involving the left liver measuring 86 × 53 mm with irregular shaggy margins (asterisk)

An abdominal CT scan was performed (Figures 1 and 2) and showed multiple confluent hypovascular nodular lesions involving the left liver measuring 86 mm * 53 mm with irregular shaggy margins. There was no enhancement in the early arterial phase and in the portal and equilibrium phases. Multiple nodular hypovascular lesions of other liver segments (V and VI) were noted. No veins or arteries infiltration has been detected. The common bile duct and intrahepatic biliary ducts were normal in size without suggestive signs of cholelitiasis. A suspect hilar adenopathy was noted.

Based on these findings, liver abscess was suspected, and the patient was given cefotaxime and metronidazole intravenously for 2 weeks and ciprofloxacin orally for 10 days. A percutaneous drainage was performed but unfortunately failed.

After more than 3 weeks of broad‐spectrum antibiotics, the symptoms were persistent and C‐reactive protein remained high (90 mg/L). His tumor markers were as follows: alpha‐fetoprotein (AFP) 5833.2 IU/mL, carcinoembryonic antigen (CEA) 1.9 μg/L, and CA 19‐9:5.5 IU/mL.

In view of nonamelioration of symptoms and elevated level of AFP, another CT scan was performed and showing an increasing size and number of hypodense liver lesions with peripheral enhancement.

**FIGURE 3:**
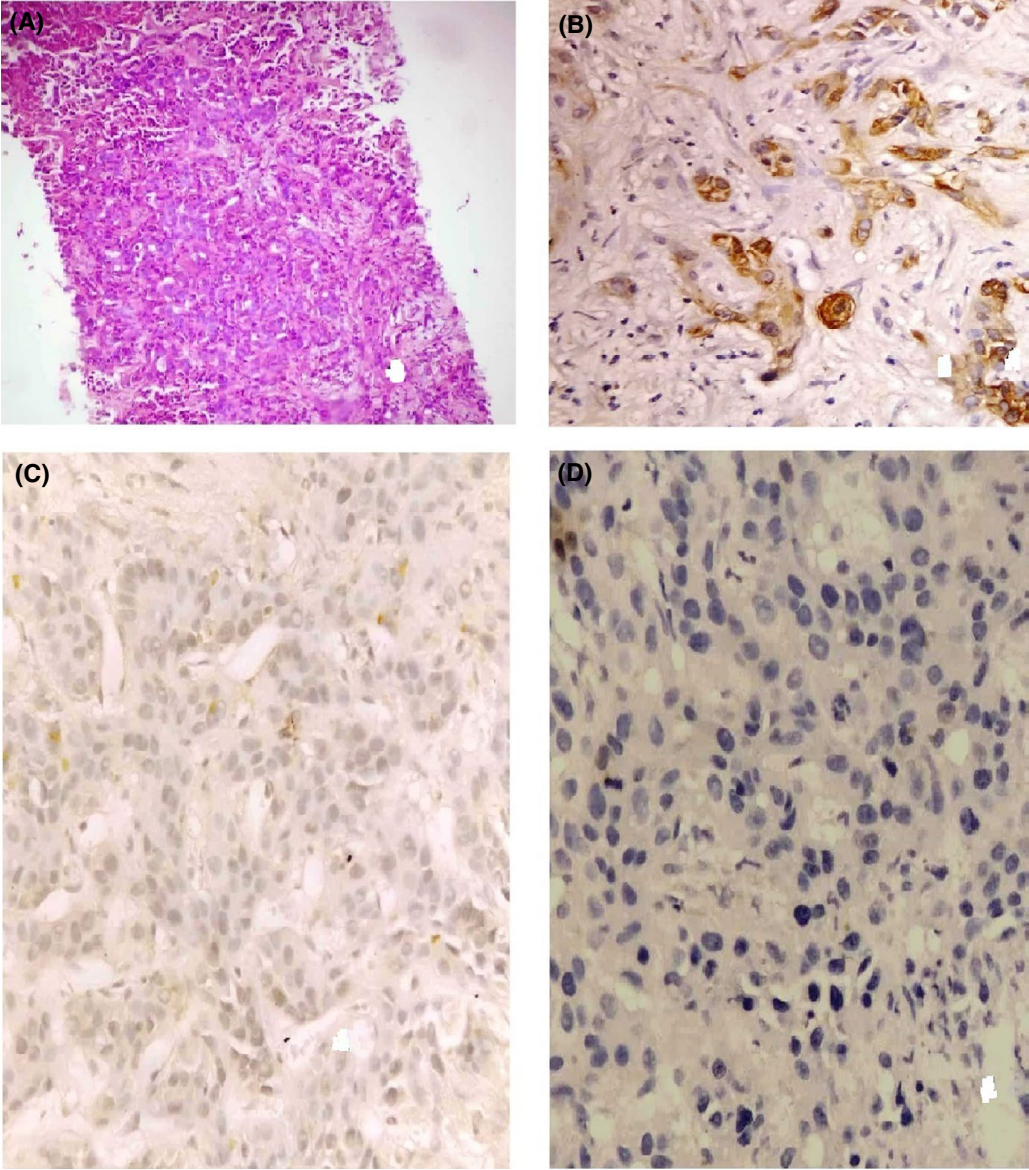
Intrahepatic cholangiocarcinoma. A, Infiltrating well‐formed glands in an abundant fibrous stroma. Malignant glands are lined by cells with varying degrees of atypia and pleomorphism. B, Positive staining for CK7. C, Negative staining for CK20 (D) negative staining for HepPar1

Because of ambiguous imaging findings, we performed a needle biopsy of the lesion that revealed large tumor cells with fairly clear limits and low cytoplasmic abundance, prominent nucleoli, arranged in a cord‐like pattern, and abundant fibrous stroma (Figure 3A). Immunohistochemistry showed positivity for CK7 (biliary origin marker) (Figure 3B). The CK20 and Hepar1 (hepatocellular origin) were negative (Figure 3C and D). Based on these immunohistochemical findings, the patient was diagnosed with ICC. CT scan of the chest showed multiple pulmonary nodules suggestive of miliary carcinomatosis of the lungs, which had not been seen in the first CT scan 3 weeks earlier. The option of surgical resection was rejected. The patient was taken care of by oncologists for chemotherapy. He died 6 weeks later.

## 3 DISCUSSION

After hepatocellular carcinoma, cholangiocarcinoma represents the second most common primary hepatic tumor.^4^ ICC is characterized by a higher incidence in man than women, late diagnosis, and poor prognosis.^5^ Established risk factors for cholangiocarcinoma are as follows: primary sclerosing cholangitis, parasitic infections, cirrhosis, hepatolithiasis, biliary duct cysts, hepatitis C virus, hepatitis B virus, diabetes, obesity, and alcohol. The distinction between ICC and extrahepatic cholangiocarcinoma is important as risk factors are different.^6^ Our patient was found to be positive for HBs antigen, and HBc and HBs antibodies with detectable viral load. ICC is classified into three types: mass‐forming, periductal infiltrating, and intraductal growth.^4^ Most observed radiological features in ICC are as follows: capsular retraction, peripheral biliary dilatation, satellite nodules, peripheral rim‐like enhancement, central delayed enhancement, and irregular tumor margins. These various radiological findings are depending on the tumor's size, location, and intratumoral components.^7^, ^8^, ^9^ A definitive pathological diagnosis is always required. Typical imaging features can easily lead to diagnose ICC. However, typical features are not always found on CT scan or MRI, and ICC may mimic a variety of nontumorous and tumorous lesions. The variable appearances of ICC pose a real diagnostic challenge.

Intrahepatic cholangiocarcinoma mimicking liver abscess is very rare. There has been no case reported till date from Tunisia. Pyogenic hepatic abscess results from a bacterial infectious process associated with destruction of the hepatic parenchyma and stroma. Escherichia coli is the most commonly isolated microorganism.^10^ The typical radiological findings are intense arterial enhancement of abscess wall and its persistence on delay phase. The center of abscess cavity usually do not enhance on any phase.^11^ If a tumor manifests necrosis, which is very common in metastatic colorectal adenocarcinoma but rare condition in ICC, it is difficult to make diagnosis without histological findings.^4^ Metastatic colorectal cancers have been first described to cause liver abscess. Pyogenic liver abscess is a rare presentation of ICC. Usually microbiological analysis shows positive bacterial growth. In our case, even if the clinical and laboratory findings suggest a liver abscess, an abscess‐mimicking lesion has been considered, but it was unresponsive to antibiotics, thus favoring a diagnosis of malignancy. Only few cases were reported in the literature.^1^, ^12^, ^13^ ICC presenting with pyrexia and leukocytosis has poor outcome.

Surgical resection is the standard treatment for cases of single ICC nodule with no evidence of metastasis.^14^ However, patients with metastases, regional lymph node metastasis, and vascular invasion are not candidate to undergo surgical resection. The present case involved a metastatic lesion. Chemotherapy was retained by our multidisciplinary team.

## 4 CONCLUSION

In conclusion, ICC mimicking liver abscess represents a difficult situation: clinical features, laboratory tests, and radiological findings could be nonspecific. Imaging modalities such as contrast‐enhanced CT scan could also be helpful in certain cases. It should be noted that in patient with liver abscess without a clear etiology, tumoral origin must be suspected. The confirmation remains histological and immunohistochemical in all cases.

## CONFLICT OF INTEREST

None.

## AUTHOR CONTRIBUTIONS

HY: concepts; HH, HY, HK, and AA: design; HY, HH, HK, and SH: definition of intellectual content; HY and HH: literature search; HY, HH, HK, and NM: manuscript preparation; HY: manuscript editing; HY, HH, NM, HK, SB, and SH: manuscript review; HH: guarantor.

## PATIENT'S CONSENT

Yes.
